# Correlations between Educational Struggle, Toxic Sites by School District and Demographic Variables, with Geographical Information System Projections

**DOI:** 10.3390/ijerph20247160

**Published:** 2023-12-09

**Authors:** Junu Shrestha, Raihan K. Khan, Shane McClintock, John DeGroote, Catherine L. Zeman

**Affiliations:** 1 School of Integrated Sciences, Sustainability, and Public Health, University of Illinois, Springfield, IL 62703, USA; jshre2@uis.edu; 2 Department of Health Sciences, College of Health and Behavioral Studies, James Madison University, Harrisonburg, VA 22801, USA; khanrk@jmu.edu; 3 Clinton County Environmental Health Department, Clinton County, DeWitt, IA 52742, USA; smcclintock@clintoncounty-ia.gov; 4 Department of Geography, College of Social & Behavioral Sciences, University of Northern Iowa, Cedar Falls, IA 50614, USA; john.degroote@uni.edu

**Keywords:** toxic score, individualized education plan, geographic information systems, correlation, environmental factors, multiple exposure

## Abstract

This correlational study associated data on children enrolled in individualized educational plans in their K-12 schools (IEP) and an algorithm-calculated score of neurotoxins at contaminated sites located in each school district. The study also mapped and projected the correlations using Geographical Information System (GIS) technology. These data were populated in ArcMap 10.5 (a GIS software) for generating maps and data to conduct geospatial analysis. A total of 1 Superfund site and 39 CERCLA sites were identified as contaminated sites for this analysis. The majority of contaminants were heavy metals such as lead, arsenic, mercury, and cadmium. The mean toxic score of all contaminated sites combined was 13.4 (SD 14.4). Correlational analysis between the IEP numbers from each school district and toxic scores from the contaminated school district sites exhibited a positive relationship (F = 23.7, *p* < 0.0001). Correlations were also seen among higher toxics scores, IEP numbers, and children under the age of 10 (*p* < 0.00052) as well as higher proportions of black students in areas with high toxics scores (*p* = 0.0032). Black students were also far more likely to be enrolled in an IEP (*p* < 0.0001). Household income and poverty percentage in contaminated areas were also correlated (*p* = 0.0002). Individuals without college degrees were overrepresented in high toxic score school districts (*p* < 0.0001). The important low socio-economic status indicator of free and reduced lunch programs also correlated with increasing toxic scores (*p* = 0.0012) and IEP numbers (*p* = 0.0416). This study emphasizes the need to account for multiple exposures to wholistically appreciate environmental factors contributing to negative health outcomes.

## 1. Introduction

Historically, the US has seen many environmental injustices toward indigenous populations, people of color, and people living in poverty [1]. Environmental and social injustice towards minoritized and “othered” groups has led to higher pollutant exposure and worse health consequences among these groups. Women and children in minoritized and/or low-income neighborhoods have suffered especially [1,2,3]. Research and advocacy beginning in the late 1980s and extending over more than 30 years and 100s of studies have established the strong association between disproportionate exposure to environmental toxins/risks and being a member of a black, indigenous, other people of color, BIPOC, or low-income status group [4]. This correlational study further contributes to the body of research identifying these relationships and indicates the need for more analytical and “in vivo” studies.

Environmental pollution gravely affects children’s health due to developmental considerations, age of exposure, and the ability of children’s bodies to detoxify exposures [5]. Children are more vulnerable to some chemicals than adults as their higher percentage of body water and higher surface-to-body ratio can mean disproportionately heavy exposure to pollutants in a given environment. Their rapidly developing cells, tissues, and organs impart a critical biological sensitivity due to growth and development [2,6]. Furthermore, infants’ abilities to detoxify environmental exposures in the first year of life are not as well developed as adults or children older than one year of age [7]. Research shows that environmental pollution can have multiorgan, complex adverse effects on children’s health and well-being from birth throughout childhood [8], such as adverse birth outcomes, higher mortality, neurological and behavioral abnormalities, respiratory disorders (e.g., asthma), childhood obesity, and cancers [2,8,9,10]. Additionally, the costs of waste generation and management seldom include the lead-on effects of health issues over time, which are costly and are borne by afflicted individuals and the public in general, not the companies generating the waste; in 2008, the annual cost of disease caused by environmental pollution was calculated to be approximately “$80 billion” [8].

Because of past national attention toward toxic chemical sites such as Love Canal (a neighborhood in Niagara, New York, infamous for massive environmental contamination and children’s health issues, including cancers) [11,12] and the Valley of the Drums (a 23-acre toxic site in Northern Kentucky where over 100,000, 55-gallon drums of toxic, hazardous waste had been discarded into pits on the ground and left in barrels strewn about the site) and on-going research and social justice revelations [13,14], the US Congress established the Comprehensive Environmental Response, Compensation and Liability Act (CERCLA) in 1980, which is informally called “Superfund” and is the US law that deals with locating, classifying, and responding to abandoned hazardous waste sites [14]. Superfund sites are cleaned up by the EPA (or their contractors) [15]. There are currently 1887 superfund sites under clean-up actions in the US [16]. Brownfields are contaminated industrial properties that may or may not qualify as Superfund sites, the expansion, redevelopment, or reuse of which might be complicated by the presence or potential presence of hazardous pollutants, substances, or contaminants [17,18]. Although the EPA established the Brownfields and Land Revitalization Program in 1995 [17], it does not clean up brownfield sites [19]. The responsibility of cleaning up and managing brownfield sites is on state government entities and non-profit organizations through competitive grants [19]. There are approximately 450,000 brownfield sites in the US [17]. Brownfield locations are often public health concerns. Communities near brownfields experience safety risks, social and economic struggles, and environmental health issues [20]. Common brownfield contaminants include arsenic, asbestos, lead, petroleum hydrocarbons, polycyclic aromatic hydrocarbons (PAHs), polychlorinated biphenyls (PCBs), volatile organic compounds, cadmium, chromium, dioxin, mercury, and other pollutants [21]. These contaminants are highly harmful to human health, especially children’s health, and many are known carcinogens and neurotoxins. For example, long-term arsenic exposure can lead to cancer, skin changes, and organ failure (e.g., liver and kidney failure) [22]. Long-term asbestos exposure can cause asbestosis and lung cancers, including mesothelioma (cancer of the pleural lining of the thoracic or abdominal cavities) [23]. Lead exposure in children can cause anemia, reduced growth, and neurological damage, leading to low IQ and hyperactivity, hearing and behavior, and learning problems [24].

Pregnant women’s exposure to lead is an example of intergenerational impact. It can lead to serious developmental anomalies in their fetuses and infants [24]. Therefore, examining the potential exposure patterns and health effects on children living near brownfield sites, particularly sites where known neurotoxins are present, is important. This correlational analysis examines the strength of the association between known brownfield sites as geolocated in school districts and the number of Individual Educational Plans IEPs in those districts. This study aimed to correlate the potential for exposure to brownfield contaminants in the school district with children’s developmental health, using IEPs as a proxy for their learning challenges. IEPs are not a measure of IQ but plans that are developed when children struggle to keep pace in school with their peers and need additional, tailored services to make good progress. This study also correlates demographic variables such as race, educational attainment, and the need to rely on reduced-cost/low-cost lunch programs as a proxy for socioeconomic impacts with toxic scores from the school district brownfield sites to examine the patterns of these relationships. The demographic variables, including a proxy measure of socioeconomic status, were essential as they are effect modifiers that function with environmental exposures [25]. To the authors’ knowledge, no such study has ever been conducted in Black Hawk County, Iowa (IA). This paper will describe the methods used to locate brownfield sites, identify toxins, and develop an algorithm to numerically quantify the dangers at the sites, identify geospatial data, and correlate these data statistically and geospatially. The conclusions will discuss the strengths and weaknesses of the work and suggest additional work and the need for this work to be completed at the county/regional health department level to monitor populations for possible impacts and mitigate possible future implications of exposure.

## 2. Materials and Methods

### 2.1. Correlational Study Design

This study utilizes a correlational study design. Preexisting data were correlated in the areas of demographics, number of children enrolled in IEPs within school districts, and a toxics score derived from calculations applied to data about the 40 contaminated sites in the study area. As a correlational study, individual exposures and individual outcomes are not examined, and its findings are hypothesis generating, not hypothesis testing. Nevertheless, correlational studies play important roles in stressing relationships that deserve additional study.

### 2.2. Locating Contaminated Sites

**Figure 1:**
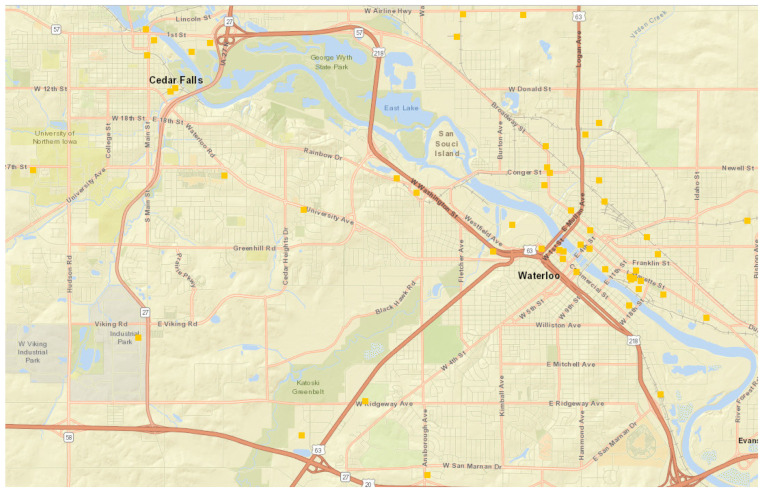
Contaminated sites as listed in Iowa DNR Facility Explorer Website.

Brownfield sites were identified using the Iowa Department of Natural Resources (IDNR) “Facility Explorer” website. The State of Iowa and the Iowa Department of Natural Resources classify CERCLA and brownfield sites together as “contaminated sites,” which is used interchangeably with CERCLA and brownfield sites in the rest of this paper. The State of Iowa and the Iowa Department of Natural Resources address these contaminated sites under the Land Recycling Program, which includes chapters 133 and 137 of the Iowa Administrative Code, to enforce cleanup responsibilities to those responsible for the environmental concerns or those that have assumed responsibility thereafter. This publicly available website lists all contaminated sites and contaminants present, including soil and water samples that were tested to quantify any possible level of contamination (Figure 1). Thus, study sites were identified, along with the number and type of contaminants present at each site and the number of years the site had been under management. After site identification, the latitude and longitude of each site were obtained from an IDNR Facility Explorer website [26]. The sites were selected based on criteria of being a superfund site, brownfield site, CERCLA pre-remedial site, CERCLA remedial site, a site registered as Iowa Chapter 133/Chapter 137 program, or an RCRA site. This allowed researchers to identify a total of 40 Sites, with 35 being used for analysis due to 5 of the sites having incomplete information regarding contaminants.

### 2.3. Demographics Data Collection and Sources of IEP Data

The data on demographics such as age, education, race/ethnicity, income, and poverty percentage were taken from the United States Census Bureau 2015 census data. The study also included data on the counts by the school of individualized education plans (IEP) obtained from Cedar Falls and Waterloo Community School District Area Education Agency, AEA offices, in the Spring of 2017. AEA offices declined to provide demographics beyond the number of IEPs per school demarcated by grade. Private schools in the districts were contacted but declined to participate in the study. Data on students enrolled in free or reduced-cost lunch programs in each school district were obtained from the Iowa Department of Education 2016–2017 database.

### 2.4. GIS Data Collection Sources: Toxics Sites, School District Polygons, Housing Characteristics

Toxic sites needed to be precisely geospatially located, data identifying the geospatial boundaries of school districts were needed, and age of housing data were sought (an important indicator of possible lead exposure within the home). These data would be used both statistically and geospatially to correlate with the number of IEPs in the respective school districts. The coordinate of each contaminated site was fixed using a GPS device. The polygon (a shape file recognized by ArcGIS associated with a data spreadsheet for unique data points contained within that shape file) feature classes of the Waterloo and Cedar Falls school districts were obtained from the “locate my school” website (https://www.locatemyschool.com/map/waterloo, accessed on 15 July 2017). It is an interactive web-based mapping format that shows a school district boundary map.

### 2.5. Embedding Contaminated Sites with School District

A polygon feature class was created for each contaminated site by carefully aligning the boundary with google maps. The geoprocessing tool intersect was used to embed contaminated sites with school districts. In some cases, one school district consisted of multiple contaminated sites. Therefore, the toxic score of multiple contaminated sites was added together for one school district.

### 2.6. Calculating Toxic Score

Each contaminated site was evaluated for three categories of chemicals: heavy metals, polyaromatic hydrocarbons (PAH), and solvents. These chemical categories were chosen for their known neurotoxic effects, which have been well documented, and for their ability to remain for many years in the environment [27]. It has been well established that metals, polyaromatic hydrocarbons, and solvents in general (as broad classes of petroleum-derived solvents) are neurotoxic [28]. The metals are elements that do not degrade, are known to be very neurotoxic, and have been found to drift in dust in the air for up to 8 miles. Thus, they received a score of 1 (x = each instance per site); PAHs degrade, but more slowly than solvents, so they received a score of 0.5 (x = each instance per site), and solvents received a score of 0.25 (x = each instance per sites) as they are most likely to degrade if found in the soil at depths less than 3 feet (after 3 feet, solvents and PAHs can still be detected on orders of decades). Water, soil disturbance, etc., can bring these PAHs and solvents to the surface, where they can become distributed with air particles or are encountered on or around sites [29,30]. Sites scores within school districts were summed to determine a district score.

The toxic score, TS, of the individual brownfield sites, was determined by weighting each of the contaminants present at each site on a scale of 0–1 and summing them. Based on the level of neurotoxicity as supported by previous toxicological studies, the weighting factor used was: heavy metals—1, PAH—0.5, and solvents—-0.25. Thus, a numeric formula for the algorithm can be represented as:

TS = (Xhm1 (x1 + x2…) + Ypah0.5 (y1 + y2…) + Zslv0.25 × (z1 + z2…))(1)

where TS = toxics score; hm = heavy metal; pah = polyaromatic hydrocarbon; and slv = solvent.

If the contaminated sites had multiple chemicals present, each of those chemicals and each of the chemical categories was summed to derive the toxic score. For example, if the site had contaminants such as arsenic, lead, dibenzo pyrene, and toluene, then the toxic score of that contaminated site for characterizing its neurotoxic weighting is 1 + 1 + 0.5 + 0.25 = 2.75.

Python script was used to automate the task of generating the toxic score weighting of each contaminated site based on the above algorithm and the identified contaminants at the site. The chemicals present and their weighting factors are provided in Table 1.

### 2.7. GIS Tools and Data Analysis

Statistical analysis using JMP version 14 and GIS tools were used to explore correlations between the toxic score for the smallest unit of geographical measurement (elementary districts included in the larger middle and high school districts) and IEP data across all school sites. 

The GIS tools used during the research were mapping overlay tools such as intersect, buffer, and union. We also used a Python script [31] for repetitive analysis to add the toxic score in each school district. The table analysis and management tools were used to join the existing Excel sheets [32] and geocoding for mapping techniques. After using GIS overlay tools, the final attribute table was imported, and data were analyzed using JMP, Statistical Discovery software, Cary, NC, USA [33].

## 3. Results

### 3.1. School-Based Demographics, IEP Data, and Associated Contaminated Sites

There were 26 schools at all levels in the sample from which IEP data were derived. A total of 65% of the schools (*n* = 7) were located in Waterloo School Districts, while 35% of the schools (*n* = 9) were located in the Cedar Falls-associated school districts. The majority of the sample consisted of elementary schools (17, 0.65), then middle schools (6, 0.23), followed by high schools (3, 0.12). The number of toxic sites per school district was impacted by the school type (elementary, middle, or high school) as districts enlarged with higher grades. The mean number of toxic sites within the school district ranged from 0 for three Waterloo and two Cedar Falls districts to a high of 15 in one Waterloo High School district. The mean number of sites per school district was 3.6 (sd 3.58). The algorithmically derived toxic score ranged from 0 to 65.25 (the high number associated with the Waterloo High School district with 15 toxic sites within the district). The mean toxics score was 13.4 (sd 14.4). IEPs ranged from a low of 41 to a high of 247, with a mean of 85.3 (sd 49.8).

The percentage of minority students ranged from a low of 4.9% to a high of 79.5%. The percentage of multi-racial students ranged from a low of 3.1% to a high of 13.6 percent. Table 2 indicates the percentage of youths receiving and eligible for free or reduced-cost lunch as a proxy for food scarcity and socioeconomic status, indicating a low of 12% eligibility in one Cedar Falls elementary school district and a high of 95% eligibility in one Waterloo elementary school district. These two percentages of eligibility and students actually receiving free/reduced lunch are in agreement 57.7 percent of the time. See Table 2 for a coded listing of schools and these data points. The authors have coded the original school names to protect their identity and to reduce stigma.

### 3.2. Contaminated Sites

**Figure 2:**
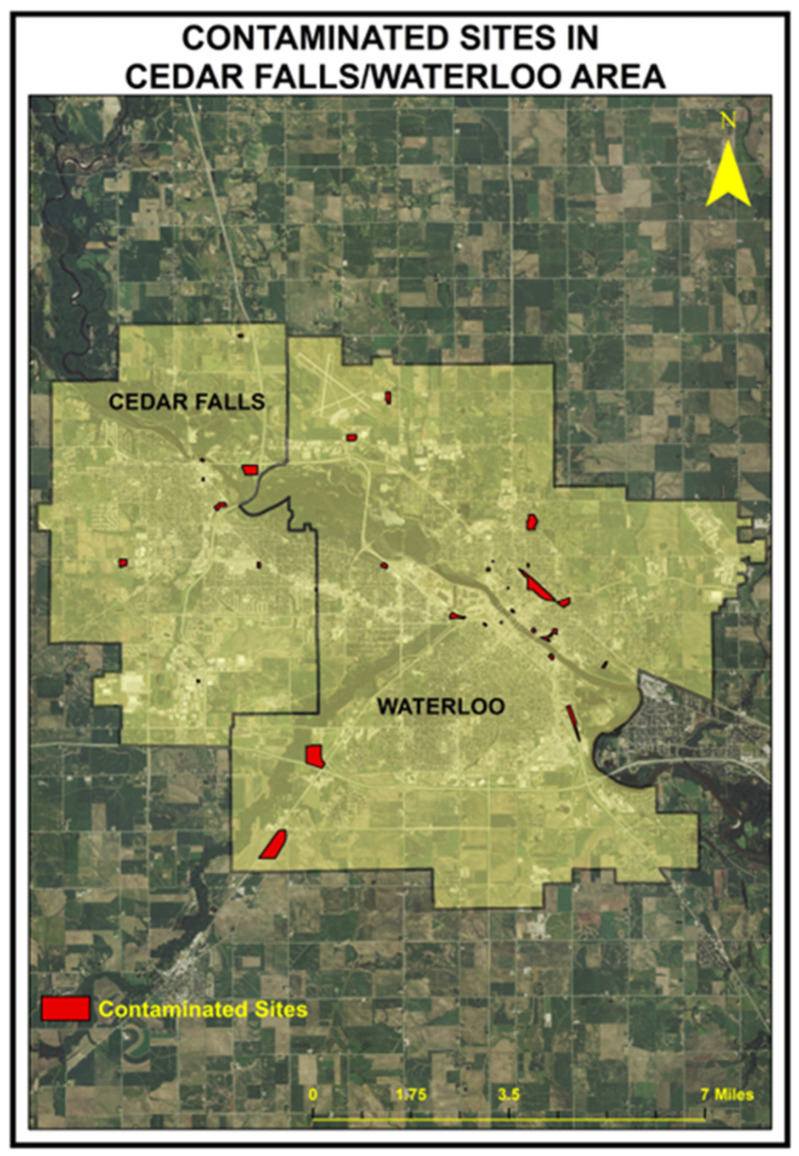
List of contaminated sites.

**Figure 3:**
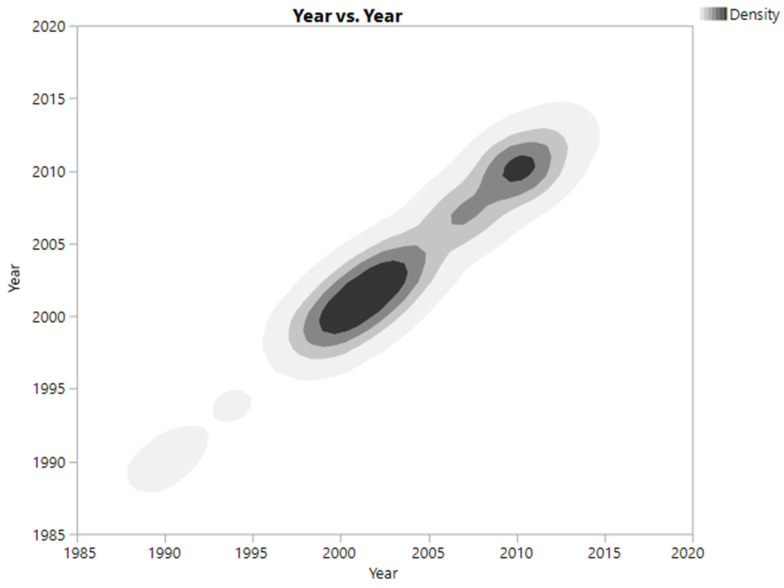
Year of first testing vs. density of time of sites.

The figure below shows the contaminated sites in the red polygon (shape), Figure 2. The date of first testing of the contaminated sites ranged from 1989 to 2013, with a median date for all sites tested of 2003, 14 years before the IEP data were compiled. Figure 3 is a density plot of the time of first testing over the time period sites were discovered. As can be seen, the majority of all sites (darkest concentrations) were known and tested before 2010.

**Figure 4:**
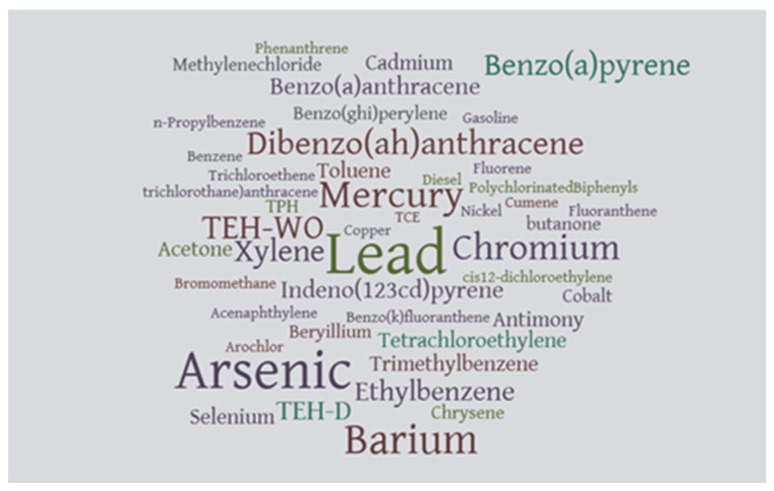
Constellation of contaminants.

A qualitative analysis using a word cloud indicated that heavy metals were frequently reported in these contaminated sites, such as lead, arsenic, mercury, and chromium. Figure 4 below shows the constellation of contaminants in the study area.

### 3.3. Toxic Scores and IEP Statistical Analysis

School locations, school district polygons, and the correlational relationship between toxic scores and IEPs by school were explored. Bivariate analysis between the total toxic score and total IEP numbers was calculated based on the smallest unit of physical proximity, the elementary school district. Groupings of middle school and high school districts were drawn from the existing elementary groups.

**Figure 5:**
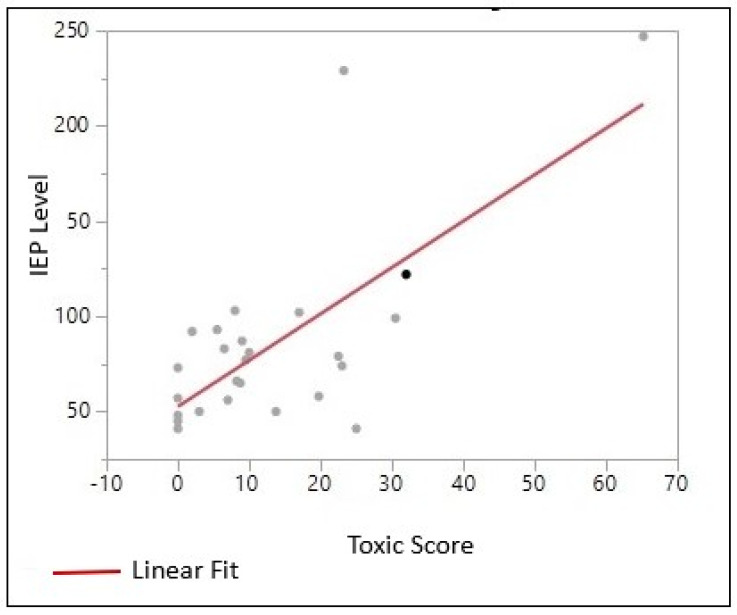
Correlation between toxic score and IEP numbers. IEP numbers = 52.56294 + 2.4359472 × Toxic Score.

The figure below shows an increase in IEP numbers, an increased toxic score, and a clear linear line between those parameters by bivariate fit and analysis of variance. The mean variance of the number of students enrolled in the IEP program is approximately 24 (23.7027) times greater, with an increased toxic score. The *p*-value was <0.0001, showing a significant correlation (Figure 5, Table 3).

### 3.4. Demographics of Race and Poverty Proxy

#### 3.4.1. Age by Toxic Score

Population demographic data (2015 US Census) of children under nine years of age were correlated with the toxic scores by the smallest unit of district demarcation (elementary). The result showed a mild correlation with children under 10 living in areas with a high toxic score (Figure S1, Table 4).

#### 3.4.2. White vs. Black Population by Toxic Score and IEP

The study also found a correlation between the black and white populations by toxic score. The study found fewer white people residing in an area with a higher toxic score (inverse relationship, downward sloping mean fit), but the scenario differs for the black population. The analysis showed a higher number of the black population residing in areas with a high toxic score. Both correlations were statistically significant (Figure S2, Table 4).

Similarly, the children enrolled in IEP programs were predominantly among the black population (Figure S3, Table 4), but no significant correlation was found with the white racial/ethnic group.

#### 3.4.3. Household Income and Poverty Percentage by Toxic Score

The bivariate fit between the socioeconomic status indicators of household income and poverty percentage presented a significant correlation, indicating that people with lower household income and the highest poverty percentage resided in areas with higher toxic scores. The median household income for the area was USD 48,007, but the majority of low-income households were found to be in areas with a toxic score of more than 20 (Figure S4, Table 4).

A similar strong correlation was seen between poverty percentage and toxic score. The areas with high poverty percentages also had a high toxic score, indicating that people living in poverty resided in areas with higher toxic scores (Figure S5, Table 4).

#### 3.4.4. Education and Toxic Score

A bivariate fit between education level and toxic score showed a significant correlation between the two variables. Four categories of education level were available: no degree, high school, some college degree, bachelor’s, and postgraduate. The analysis showed that people with a higher level of education tend to live in areas with lower toxic scores (Figure S6, Table 4).

#### 3.4.5. Free and Reduced Lunch Enrollment by Toxic Score and IEP numbers

A bivariate fit with students eligible to enroll in free or reduced lunch programs significantly correlated with the toxic score and IEP numbers. Figure S7 shows the increasing trend of eligible children enrolling in free and reduced lunch programs with increasing toxic scores and IEP numbers. Table 4 also shows the significant level of correlation between these parameters.

### 3.5. GIS Visuals on IEP, Toxic Score, and Demographics based on school districts

#### 3.5.1. Toxic Score and IEP Numbers with Poverty Percentage

**Figure 6:**
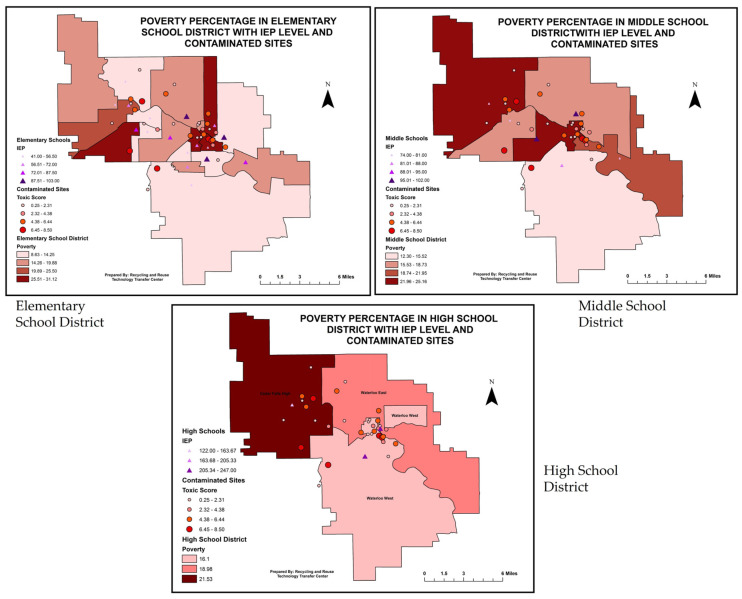
GIS visualization of poverty percentage with toxic score and IEP numbers in each census tract by elementary, middle, and high school district (*p* = 3.4.2. IEP and toxic score with black population.

GIS mapping included three layers of datasets. The first layer was the census data on poverty percentage. The second and third layers showed each school district’s IEP numbers and toxic scores. These data were replicated for elementary, middle, and high school districts. The intersect tool in ARC GIS Map 10.4.1 software, ESRI, Redlands, California, USA was used to extract the census tract data for each elementary, middle, and high school district in the study area. The visual GIS overlay map showed interesting patterns of toxic scores and IEP data on the poverty percentage. The darker color pattern shows high toxic scores, IEP numbers, and poverty percentages (Figure 6). The figure below shows high toxic scores and IEP numbers concentrated in the census tract with a high poverty percentage. The result was considered significant by a bivariate fit analysis between toxic score and poverty percentage (see previous data, Table 4).

**Figure 7:**
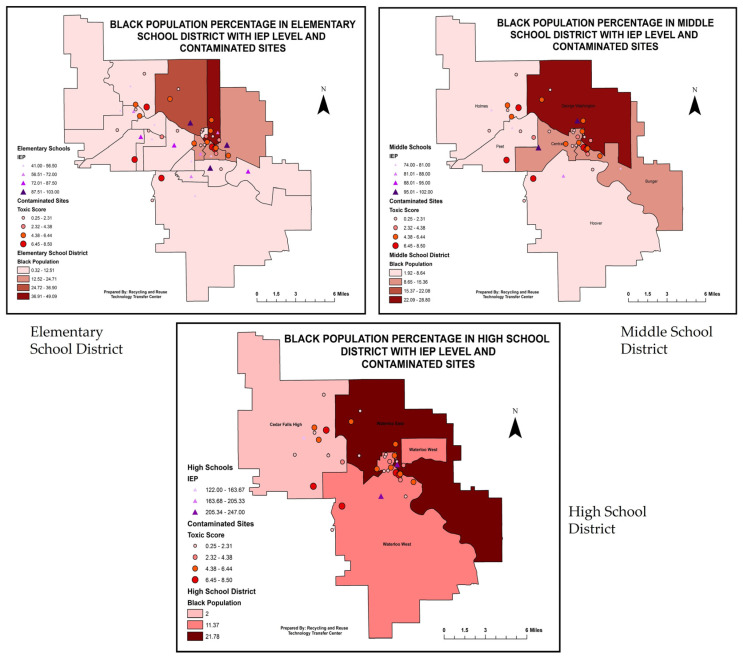
GIS visualization of black population percentage with toxic score and IEP numbers in each census tract and elementary, middle, and high school district (*p* = 0.0032 and <0.0001).

Similar to the previous figure, the GIS mapping included three layers of datasets and utilized an intersect GIS tool to extract data for the elementary, middle, and high school mapping. The first layer in this mapping was the census data on black population percentage. The second and third layers showed each school district’s IEP numbers and toxic scores. The GIS visual mapping showed that darker areas with high population percentages had high concentrations of toxic scores and IEP numbers (Figure 7). The bivariate analysis also showed a statistically significant relationship between IEP numbers and toxic scores with black population percentage (Table 4).

#### 3.5.2. IEP and Toxic Score with Free/Reduced Lunch in Elementary School District

**Figure 8:**
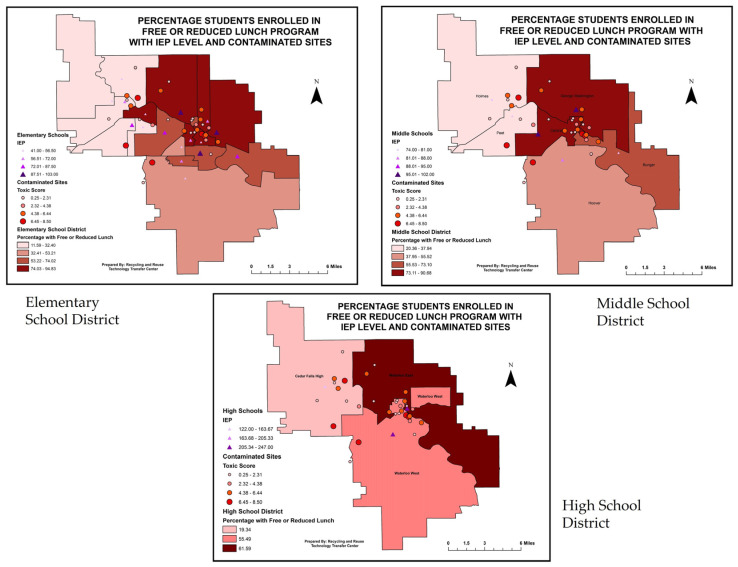
GIS visualization of students enrolled in free/reduced lunch program with toxic score and IEP numbers in each elementary school district, (*p* = 0.0416).

Again, with the assistance of an Intersect GIS tool extracting data for the elementary, middle, and high school sites, this map shows the visual graphics in three layers: percentage of students enrolled in free or reduced lunch program (shown in darker polygon), toxic score (shown in circle), and IEP numbers (shown in triangle). The map in Figure 8 shows an interesting pattern of high toxic scores and IEP numbers concentrated in school districts where the highest percentage of kids are enrolled in free or reduced lunch programs. 

## 4. Discussion

The contaminated sites in this study were examined for their neurotoxic compounds. The majority of toxic materials at the sites were comprised of heavy metals such as lead, arsenic, barium, chromium, and mercury. The sites also contained polyaromatic hydrocarbons such as benzopyrene and dibenzo anthracene. Brownfield sites are mainly contaminated with chemicals such as lead, petroleum, asbestos, polyaromatic hydrocarbons, other metals, and arsenic [34]. Most of these substances have both acute and chronic neurotoxic effects.

IEP is a program that ensures a child with identified learning challenges and disabilities attending elementary and secondary educational institutions receives specialized instruction and related services. A child can enroll in IEP based on their present level of educational performance [35]. The disability, including (or a combination of) ADHD, autism, lower IQ, and physical disability, is evaluated through the child’s progress and performance in general education.

The Iowa Department of Education, Bureau of Nutrition and Health Services uses household income as a guideline to enroll students in free or reduced lunch programs [36]. Table 4 and Figure S4 indicate that people living in an area with a high toxic score had lower household income. Tyrell et al. found that the poverty–income ratio was associated with 18 chemicals, including heavy metals such as lead, cadmium, arsenic, and mercury. Environmental exposure is mainly related to smoking and diet [25]. 

Table 4 and Figure S7 correspond to similar effects where kids enrolled in the free or reduced lunch program live in high toxic score areas. Table 3 and Figure 5 also indicate higher IEP numbers with increasing toxic scores. Therefore, the study found that socioeconomic factors such as household income, poverty percentage, education, and kids enrolled in free or reduced lunch programs are good indicators to establish a correlation between IEP numbers and toxic scores in each school district. Environmental pollutants such as lead are associated with clinically diagnosed attention-deficit/hyperactivity disorder (ADHD). According to Nigg et al., 2008, blood lead levels were statistically significantly higher in kids with ADHD than in non-ADHD control (*p* < 0.05) children. The blood lead levels were also linked with a lower IQ (*p* < 0.05) in the study population. Children exposed to environmental risk factors resulted in decrements in psychological or cognitive functioning [37].

Long-term exposure to lead in children has been linked to learning disabilities due to critical enzyme disruption and damage in the central nervous system (CNS), leading to reduced cognitive and neurobehavioral development [38,39,40]. Numerous studies report an association between chronic arsenicosis and neuro-cognitive disorders, including encephalopathy, among children [41]. Exposure to mercury in early childhood was found to be associated with lower IQ [40]. Research also shows that prenatal exposure of mothers to mercury can also lead to neuro-cognitive developmental delay and abnormality among children [42,43]. Although the effects of chromium on children’s cognitive abilities require more rigorous study, a recent study noted that prenatal exposure to chromium toxicity could reduce fetal growth, which could lead to lower IQ and increased IEP scores in children [44].

Medical doctor Rupa Marya and health sociologist Raj Patel describe the delicate balance between the neuronal structures of the central nervous system, CNS, and the supporting glial cells (about 50% overall of the CNS) that maintain that system [45]. They describe how these cells work together to support cognition and learning as a system, each being conceptualized in their work as an orchestra that must develop as a fully functional system to provide an individual with the full experience of consciousness and the ability to learn, grow, and assimilate information. They note that “when neurological systems are working well, you’re able to engage in fluent conversation, to read, to name and recall, to ponder and imagine and explore and analyze [45]”. And, they note that in the larger system of environmental, social, and racial inequities, the opportunity and likelihood of developing an exquisitely balanced and optimally functioning CNS are challenged and compromised. Is it not likely that this imperiled opportunity will lead to further missed opportunities and milestones? That this legacy of exposure and compromise have lead on cascade effects that put young, developing minds at risk? When does compromised learning translate into missed educational opportunities that lead to missed economic opportunities? When could those challenges lead to acting out as a juvenile, leading to police involvement, which disproportionately occurs in minority and low-income communities? In a sample of juveniles in detention in Connecticut, researchers found that learning disabilities in 1337 detained juveniles ranged from 13 to 40% using two different instruments (Wide Range Achievement Test and a computerized educational screener) with an average of 24.9% for the total sample, much higher than the average population [46].

It should be remembered that correlational (ecological) studies are complicated by the fact that data sets are correlated at a group level. The nature of such studies precludes the association of individual exposure with individual outcomes. This kind of study suggests hypotheses that need to be further tested and proven and is not definitive proof of a particular exposure’s impact on health outcomes. However, it is a strong correlational analysis, and it points to the need for further cohort or case-control studies that can establish both the timeline of exposure and outcome and the strength of that individual association. It rightly asks us to question and further explore the physical, environmental, social, and equity structures of our communities that may be creating and perpetuating inequities.

## 5. Conclusions

This study found that youths experiencing behavior and learning difficulties enrolled in individualized education plans within their respective school districts were more likely to live in areas with higher toxic scores, as determined by the number of CERCLIS-listed sites in those school districts and the number and types of neurotoxic substances reported to be present at those sites (Figure 5, Table 2). Also, families with lower incomes lived in school district areas with higher toxic scores (Figure S4). When all these variables were visualized using GPS/GIS applications, the areas with a higher poverty percentage along with higher toxic scores illustrated a preponderance of youths enrolled in IEPs in their school districts (Figure 6). In a study that looked at three separate states on students from low-income families and special education, the authors found that students from low-income backgrounds in all three states were disproportionately placed in special education and more than twice as likely to be put in substantially separate classrooms, compared to their non-low-income peers [47].

The positive correlation between racial ethnic minority populations (African American, Native American, Hispanic, etc.) with toxic scores and IEP indicated that racial-ethnic minority youths living in school districts with high toxic scores were more likely to be enrolled in IEP plans. The representation of students of color in special education is a concern, especially those coming from low-income families. Black students, for example, are twice as likely to be labeled as emotionally disturbed and three times as likely to be identified with intellectual disabilities compared to their white peers. Also, one in four black boys with disabilities is suspended each year, compared to only one in ten white boys with disabilities [48]. Further, Native American students receive special education services 53% more than Anglo students and receive services for developmental delay 189% more than Anglo students [49]. Special education researchers point to the multiple factors that may be influencing these outcome disparities in the need for special education services, including poverty, teacher perception, and sociohistorical context [50]. This study makes a strong case that environmental factors and exposures should be considered also. And it indicates the need to develop sophisticated multiple-exposure models and studies that can correlate individual exposures with individual outcomes. It is only by truly understanding the fundamentals of inequity in the environment and exposure that a full understanding and elimination of environmental inequity can be achieved.

## Figures and Tables

**Table 1:** List of chemicals with weighting factors found in black hawk county brownfields data.

Metals (WF = 1)	PAH (WF = 0.5)	Solvents (WF = 0.25)
Antimony, arsenic, barium, beryllium, cadmium, chromium, cobalt, copper, lead, mercury, nickel, selenium	(124-Trimethylbenzene), (135-Trimethylbenzene), Acenaphthylene, Benzene, Benzo(a)anthracene, Benzo(a)pyrene, Benzo(b)fluoranthene, Benzo(ghi)perylene, Benzo(k)fluoranthene, Chrysene, Cumene, Dibenzo(ah)anthracene, Diesel, Ethylbenzene, Fluoranthene, Fluorene, Gasoline, Indenol(123cd) pyrene, Phenanthrene, Polychlorinated Biphenyls, TEH-D, TEH-WO, Xylene, n-Propyl benzene, Arochlor1260	(111trichlorothane) anthracene, 2butanone, Acetone, Bromomethane, Methylene chloride, TCE, TPH, Tetrachloroethylene, Trichloroethene, cis12-dichloroethylene, Toluene

**Table 2:** Key demographics of school sample and related toxics score.

School (Coded)	City	School Type	# Sites (in District)	Toxics Score	IEP #	% Minority	% Multi-Race	% Taking Free or Reduced Lunch	% Eligible Free or Reduced Lunch
W1	W	E	5	19.75	58	79	9	88	95
W2	W	E	4	10	81	38	11	67	72
W3	W	E	1	5.5	93	61	9	85	90
W4	W	E	2	8.25	66	65	13	89	91
W5	W	E	0	0	48	18	8	44	44
W6	W	E	1	2	92	37	10	54	66
W7	W	E	3	8	103	67	11	85	90
W8	W	E	0	0	57	39	5	59	59
W9	W	E	4	25	41	55	14	89	89
W10	W	E	2	7	56	18	10	46	49
W11	W	E	0	0	73	11	10	61	61
CF1	CF	E	2	3	50	12	7	27	27
CF2	CF	E	1	6.5	83	11	5	10	12
CF3	CF	E	0	0	41	9	6	23	24
CF4	CF	E	3	8.75	65	13	3	22	22
CF5	CF	E	3	13.75	50	5	6	36	38
CF6	CF	E	0	0	45	13	3	15	15
CF7	CF	M	6	22.5	79	12	5	22	22
CF8	CF	M	3	9.5	77	12	5	20	20
W12	W	M	4	23	74	23	6	63	63
W13	W	M	10	30.5	99	55	9	86	86
W14	W	M	6	17	102	73	8	91	91
W15	W	M	3	9	87	25	5	50	50
W16	W	HS	7	23.25	229	47	6	62	62
W17	W	HS	15	65.25	247	37	5	55	55
CF9	CF	HS	9	32	122	13	4	19	19

School coded to protect student anonymity; City = W = Waterloo, CF = Cedar Falls; E = elementary, M = middle school, HS = high school; # Sites = number of toxic sites in school district associated with that school; toxics score = algorithm derived ranking of neurotoxins present at toxics sites; IEP # = number of individual educational programs at the school; % free or reduced lunch = percentage of students actually receiving free/reduced lunch.

**Table 3:** IEP number by toxic score and total sites, *n* = 26.

Source	DF	Sum of Squares	Mean Square	F Ratio
Model	1	30,838.389	30,838.4	23.7027
Error	24	31,225.149	1301.0	Prob > F
C. Total	25	62,063.538		<0.0001

**Table 4:** Correlation between demographics and toxic score and IEP numbers.

Demographics	RSq	Mean of Response	F Ratio	*p*-Value
Age (0 to 9) and toxic score	0.064	12.48	8.10	0.0052
White population and toxic score	0.13	264.66	18.32	<0.0001
Black population and toxic score	0.07	117.73	9.03	0.0032
Black population and IEP numbers	0.24	117.73	37.70	<0.0001
Household income and toxic score	0.11	45,725.99	14.96	0.0002
Poverty and toxic score	0.045	19.30	5.53	0.0203
No degree and toxic score	0.19	11.75	28.29	<0.0001
High school and toxic score	0.109	32.56	14.33	0.0002
Bachelor and toxic score	0.19	16.35	28.52	<0.0001
Postgraduate and toxic score	0.15	8.69	21.64	<0.0001
Eligible free or reduced program and toxic score	0.08	63.22	11.068	0.0012
Eligible free or reduced program and IEP numbers	0.03	63.22	4.24	0.0416

## Data Availability

Data requests should be directed to the primary and senior author of this text.

## Supplementary Materials

The following supporting information can be downloaded at: https://www.mdpi.com/article/10.3390/ijerph20247160/s1. Figure S1: Correlation between Toxic Score and Age (0 to 9) (*p* = 0.00052); Figure S2: Correlation between Black (Left, *p* = 0.0032) and White (Right, *p* = <0.0001) Population and Toxic Score; Figure S3: Correlation between Black Population and IEP Level (*p* = <0.0001); Figure S4: Bivariate fit of Household Income and Toxic Score (*p* = 0.0002); Figure S5: Bivariate fit of poverty percentage and Toxic Score (*p* = 0.0203); Figure S6: Correlation between various education levels and Toxic Score, (*p*-value range, <0.0001–0.0002); Figure S7: Correlation between children eligible to enroll in free or reduced lunch program by toxic score (left, *p* = 0.0012) and IEP (right, *p* = 0.0416).
