# Supplementary material for: Correlations between Educational Struggle, Toxic Sites by School District and Demographic Variables, with Geographical Information System Projections
Source: Int J Environ Res Public Health. 2023 Dec 9;20(24):7160. doi: 10.3390/ijerph20247160 (PMC10742704; doi:10.3390/ijerph20247160)
Supplement: Supplementary file 1 [file ijerph-20-07160-s001.zip › ijerph-2543474-supplementary.pdf]

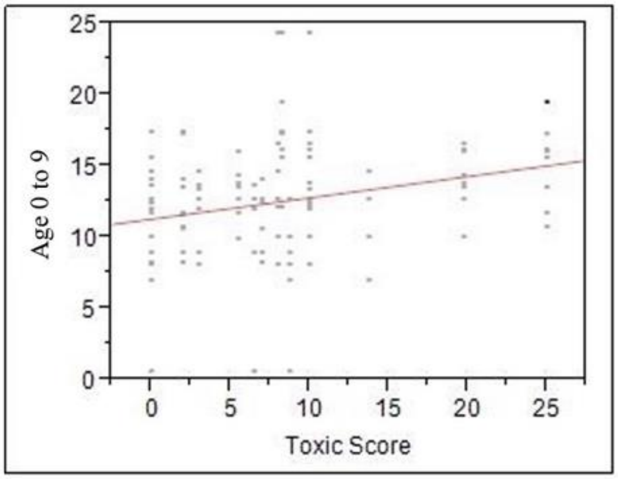

Age 0 to 9 = 11.288629 + 0.1497272* Toxic Score

Figure S1: Correlation between Toxic Score and Age (0 to 9) (p = 0.00052)

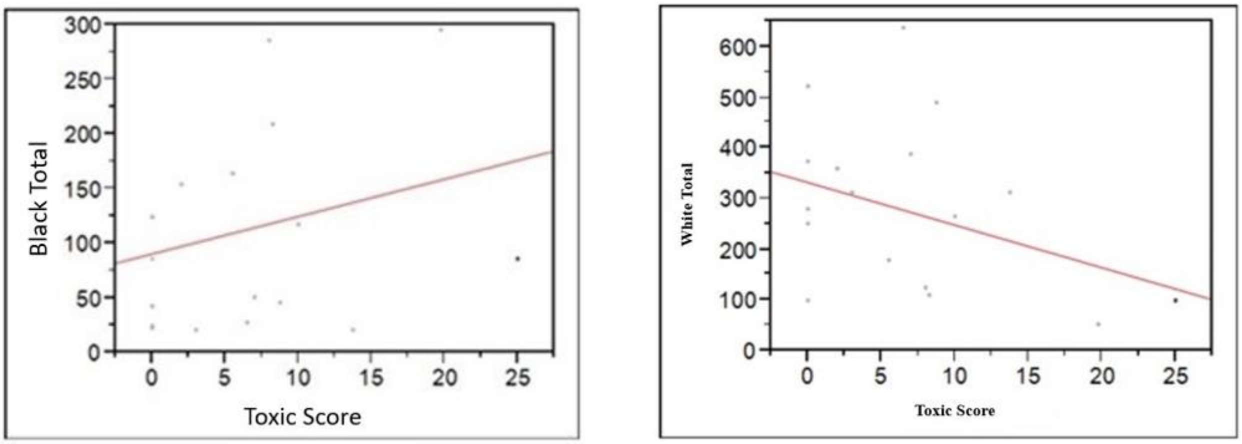

Black Total = 90.40368 = 3.4204177* Toxic Score

White Total = 331.73117 – 8.3944347* Toxic Score

Figure S2: Correlation between Black (Left, p = 0.0032) and White (Right, p = <0.0001) Population and Toxic Score

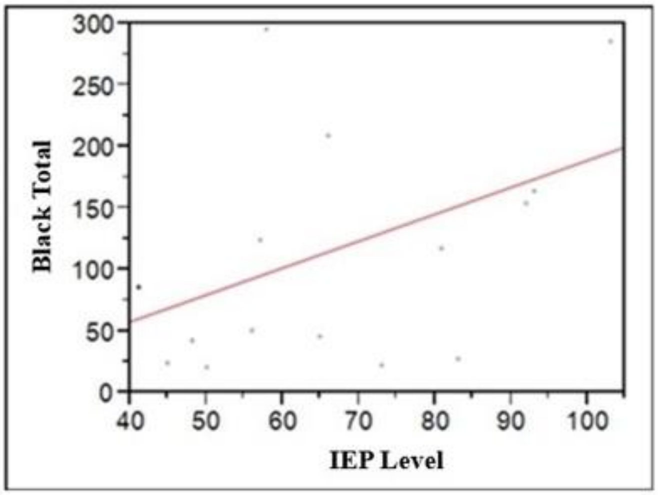

Black Total = -29.50537 + 2.1822317* IEP Level

Figure S3: Correlation between Black Population and IEP Level (p = <0.0001)

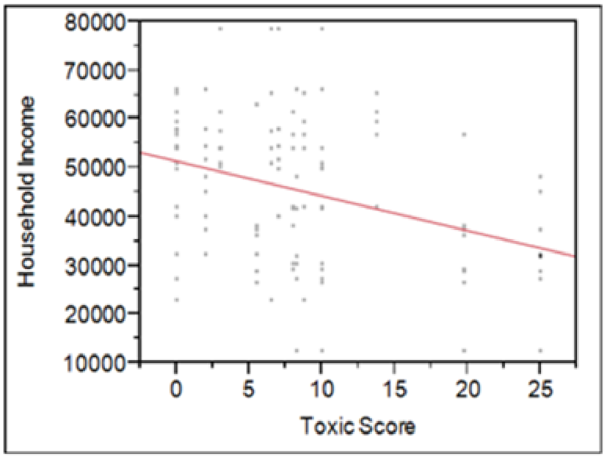

Household Income = 51422.499 – 712.99957* Toxic Score

Figure S4: Bivariate fit of Household Income and Toxic Score (p = 0.0002)

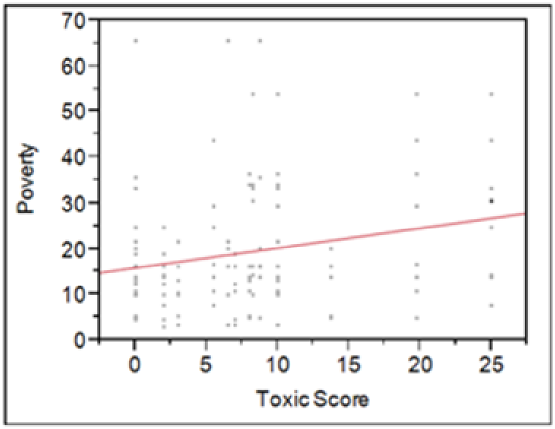

Poverty = 15.837309 + 0.4339314* Toxic Score

Figure S5: Bivariate fit of poverty percentage and Toxic Score (p = 0.0203)

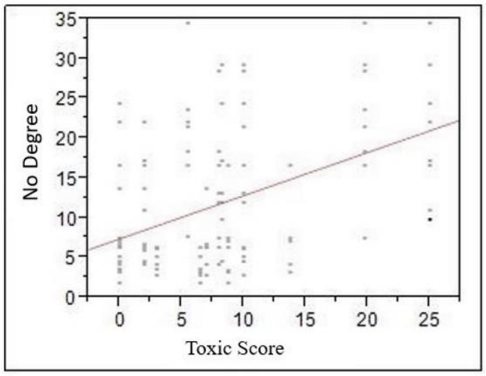

No Degree = 7.4041188 + 0.5443175* Toxic Score

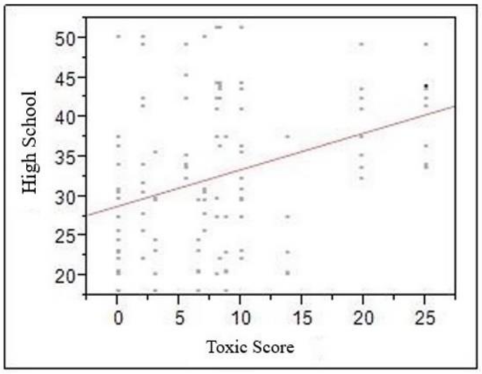

High School = 28.883622 + 0.4611612* Toxic Score

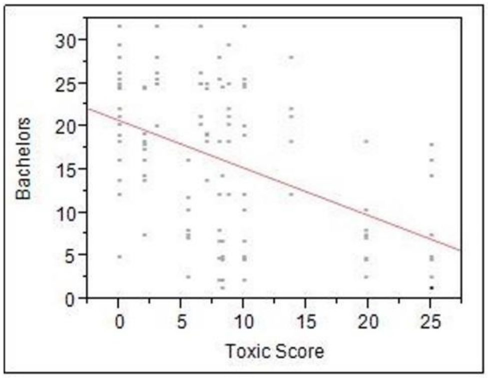

Bachelors = 20.758924 - 0.5510513* Toxic Score

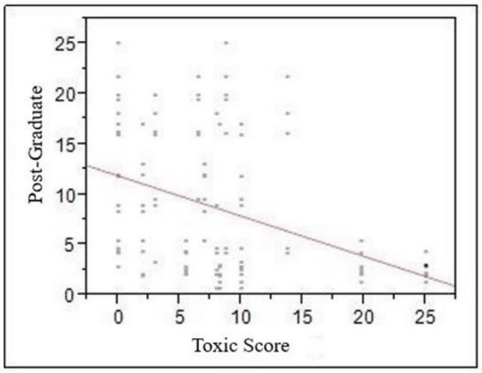

Post-Graduate = 11.897538 - 0.4013747* Toxic Score

Figure S6: Correlation between various education levels and Toxic Score (p-value range, <0.0001- 0.0002)

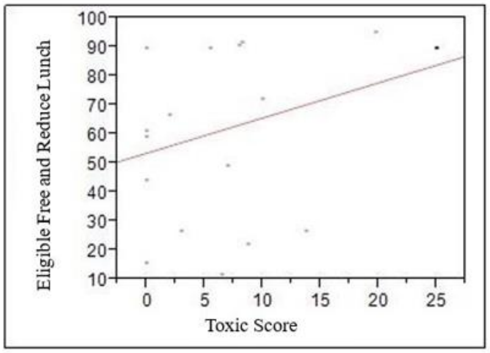

Eligible Free and Reduce Lunch = 53.53777 + 1.2124906* Toxic Score

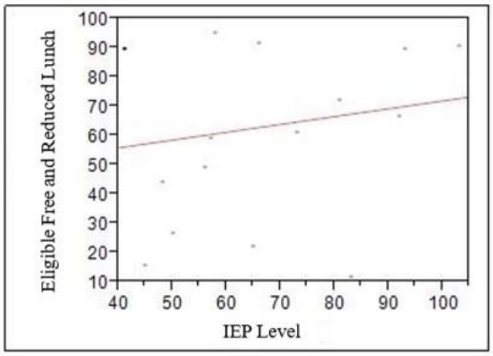

Eligible Free and Reduce Lunch = 45.217473 + 0.2668938* IEP Level

Figure S7: Correlation between children eligible to enroll in free or reduced lunch program by toxic score (left, p = 0.0012) and IEP (right, p = 0.0416)
